# Editorial: Anti-inflammatory immunopharmacology in the prevention and treatment of major chronic diseases

**DOI:** 10.3389/fphar.2023.1134918

**Published:** 2023-01-12

**Authors:** Qiuwang Zhang, Shihai Yan, Dongdong Sun, Zhirong Geng, Peixiang Zhang

**Affiliations:** ^1^ Division of Cardiology, Keenan Research Center for Biomedical Science at the Li Ka Shing Knowledge Institute, St. Michael’s Hospital, Unity Health Toronto, University of Toronto, Toronto, ON, Canada; ^2^ Department of Pharmacology, Jiangsu Provincial Hospital of Traditional Chinese Medicine, Nanjing, China; ^3^ Jiangsu Collaborative Innovation Center of Traditional Chinese Medicine in Prevention and Treatment of Tumor, Nanjing University of Chinese Medicine, Nanjing, China; ^4^ State Key Laboratory of Coordination Chemistry, School of Chemistry and Chemical Engineering, Nanjing University, Nanjing, China; ^5^ Department of Human Genetics, University of California, Los Angeles, Los Angeles, CA, United States

**Keywords:** chronic disease, inflammation, immunopharmacology, precision medicine, biomolecule

## Background

Chronic diseases (CDs) are defined by the World Health Organization (WHO) as non-communicable diseases resulting from combined actions of genetic, environmental and behavioural factors; they are of long duration and generally slow in progression (https://www.who.int/news-room/fact-sheets/detail/noncommunicable-diseases). CDs such as cardiovascular and cerebrovascular diseases, cancer and diabetes are significant contributors to death and disability worldwide. A systematic analysis for the Global Burden of Disease (GBD) Study 2015 revealed that ischemic heart disease, stroke and diabetes were the leading causes for years of life lost (GBD 2015 Mortality and Causes of Death Collaborators, 2016). A more recent study by the GBD 2019 Diseases and Injuries Collaborators, 2020 showed that in 2019, CDs were responsible for significantly increased years lived with disability, and ischemic heart disease and stroke were the top causes of disability-adjusted life-years for individuals aged 50 years and over. Using a dynamic micro-simulation model to analyze the sociodemographic factors, health behaviours, and chronic diseases and geriatric conditions of individuals ≥35 years of age to project multi-morbidity in the older population in the United Kingdom in 2035, Kingston et al., 2018 found that there would be an approximately 200% increase in cancer incidence and a 120% increase in diabetes incidence for the population aged ≥65 years of age in 2035. This data plus the fact that the global population is rapidly aging call for efforts to develop more effective interventions to prevent and treat CDs.

Inflammation has long been recognized as playing a key role in the pathogenesis of CDs (Pawelec et al., 2014; Furman et al., 2019). Inflammation is an evolutionarily conserved self-healing process involving immune and non-immune cells to eliminate pathogens and promote tissue repair and recovery. Environmental, genetic and lifestyle factors can dysregulate a normal inflammatory response, leading to chronic inflammation and eventually CDs. Despite the recognition of a close link between chronic inflammation and CDs, the mechanisms of CD pathogenesis remain to be fully understood, and more therapeutic approaches are needed.

Against the backdrop described above, this Research Topic aimed to disseminate up-to-date knowledge of anti-inflammatory immunopharmacology in the prevention and treatment of CDs.

## Articles published in the Research Topic

A total of 11 articles on basic research, clinical research or review of literature were published in this Research Topic, covering a variety of areas of anti-inflammatory immunopharmacology in the prevention and management of CDs.

Multiple sclerosis (MS), a chronic inflammatory autoimmune disease, is characterized by demyelination and neurodegeneration restricted to the central nervous system. There’s currently no cure for MS and treatments focus on controlling the condition and improving the symptoms (Hauser & Cree, 2020). In a search for anti-inflammatory molecules as therapeutic agents, Li et al. developed a recombinant flagellin protein, namely FLaAN/C, from the bacterium *Legionella pneumophila* and tested its therapeutic efficacy in mice with experimental autoimmune encephalomyelitis (EAE). They reported that FLaAN/C reduced EAE severity and repressed inflammatory cell infiltration and demyelination. FLaAN/C administration also inhibited reactive oxygen species production, upregulated anti-inflammatory cytokines, i.e., CD206, IL-10 and Arginase-1 from microglia/macrophages, and reduced levels of serum pro-inflammatory cytokines, e.g., IL-1β and TNF-α in EAE mice. Furthermore, it was demonstrated that FLaAN/C attenuated EAE by blocking NF-kB nuclear translocation and inhibiting NLRP3-mediated neuronal pyroptosis. This study may lead to the development of a novel therapeutic approach for MS.

Herbal medicine using botanical products represents an alternative, effective option for chronic disease care (Kim et al., 2018). Yang et al. explored the therapeutic potential of celastrol, an active compound extracted from *Tripterygium wilfordii,* in a rat model of osteoarthritis (OA). In cultured rat chondrocytes, celastrol was found to inhibit matrix degradation and downregulate IL-1β induced expression of inflammatory mediators, i.e., cyclooxygenase-2, IL-6 and prostaglandin E2. Cartilage tissues from OA patients and OA rats were shown to produce a substantially higher level of toll-like receptor 2 (TLR2), an essential player in inflammation. Celastrol treatment resulted in decreased osteophyte formation and bone resorption, and prevented disease progression in OA rats. Examination of mechanism of action demonstrated that celastrol abolished the up-regulatory effect of IL-1β on TLR2 in cultured chondrocytes and lowered TLR2 levels in OA rats, suggesting celastrol exerts its anti-inflammatory activity by suppressing TLR2. Vaccarin, a flavonoid glycoside extracted from *Vaccariae semen*, possesses endothelial protective activity, with the mechanism not being fully understood. Li et al. reported that vaccarin protected against endothelial inflammatory injury in type 2 diabetes mice by targeting the miR-570-3p/HDAC1 pathway. They discovered that the aorta of diabetic mice had decreased miR-570-3p and elevated HDAC1, both of which were reversed by vaccarin, leading to the alleviation of aortic inflammatory injury.

In a clinical study, Hu et al. looked into cellular heterogeneity and immunogenicity of chondrocytes in OA patients. They performed bioinformatics analysis of single-cell RNA sequencing data established using samples from 10 OA patients, and identified subtypes of chondrocytes. According to the gene expression profiles, chondrocytes from OA patients were divided into 8 subgroups: cartilage progenitor cells, effector chondrocytes (ECs), fibrocartilage chondrocytes (FCs), homeostatic chondrocytes, hypertrophic chondrocytes, prehypertrophic chondrocytes, proliferative chondrocytes and regulatory chondrocytes. Functional analyses revealed that these subtypes of chondrocytes played different roles in OA pathogenesis, with ECs and FCs possessing strong immunogenicity and being associated with disease severity. Furthermore, 6 genes highly expressed in ECs and FCs, i.e., CHRDL2, DSC2, COL1A1, COL14A1, IFI27, and THY1 were found to be valuable biomarkers for early diagnosis of OA. Additionally, findings of this study may help drive more personalized care for patients with OA.

Several review articles were published in this Research Topic. Liu et al. addressed the Research Topic in cancer chemo-immunotherapy. The long-term anticancer immune activity of chemotherapy is often restricted by the immunosuppressive tumor microenvironment. These authors presented the current knowledge of immunogenic regulatory function of chemotherapeutic agents (CTAs) and illustrated the strategies to improve CTA immunomodulatory effect. Finally, the authors suggested more research to better understand the mechanisms underlying the immunomodulatory effects of chemotherapy and identify biomarkers that can be applied to guide immunogenic chemotherapy. In another review paper, Xie et al. summarized the studies of traditional Chinese therapy (TCT) in treating sleep disorders, and concluded that the current literature supported that TCT was effective in relieving anxiety, lowering depression and improving sleep by inhibiting inflammatory reactions. They proposed TCT for the management of sleep disorders caused by COVID-19.

In summary, this Research Topic primarily offers information on mechanisms of CDs that may help improve early diagnosis and promote precision treatment, as well as exploration of biomolecules for disease treatment that could lead to the development of novel therapeutic agents for CDs.
